# Rehabilitation Workforce Challenges to Implement Person-Centered Care

**DOI:** 10.3390/ijerph19063199

**Published:** 2022-03-08

**Authors:** Júlio Belo Fernandes, Diana Vareta, Sónia Fernandes, Ana Silva Almeida, Dina Peças, Noélia Ferreira, Liliana Roldão

**Affiliations:** 1 Escola Superior de Saúde Egas Moniz, Caparica, 2829-511 Almada, Portugal; soniabelo@sapo.pt (S.F.); dinabaiao@sapo.pt (D.P.); 2 Grupo de Patologia Médica, Nutrição e Exercício Clínico (PaMNEC)—Centro de Investigação Interdisciplinar Egas Moniz (CiiEM), 2829-511 Almada, Portugal; 3 Centro Hospitalar de Setúbal, 2900-182 Setúbal, Portugal; diana_vareta@hotmail.com (D.V.); anasilvalmeida@gmail.com (A.S.A.); noelia-sf@hotmail.com (N.F.); liliana.roldao1@gmail.com (L.R.)

**Keywords:** barriers, facilitators, person-centred care, rehabilitation, qualitative research

## Abstract

There is an increasing emphasis on developing person-centered care in rehabilitation settings. However, this care practice has not been fully implemented due to several factors. This study explores rehabilitation workforce perspectives on the barriers and facilitators to implementing person-centered care (PCC). This was a quantitative descriptive study, which was developed based on interviews with 12 healthcare professionals from a private institution in the region of Lisbon and Tagus Valley in Portugal. The recruitment was made in October 2020. Braun, Clarke, Hayfield, and Terry’s content analysis was applied to the transcripts, and these were transcribed verbatim. The consolidated criteria for reporting qualitative research (COREQ) checklist were applied to this study. Participants described barriers such as an unsupportive organization and leadership, staff constraints, heavy workload, and resistance to change. Unique to this study, a patient’s clinical characteristics were identified as barriers to person-centered care. As facilitators, they described leadership, staff satisfaction, a positive physical environment, training and education, and shared decision-making. It is essential to understand the perceptions of the rehabilitation workforce, as they play an integral role in providing PCC. This study serves as a guide to facilitate person-centered care, as it provides an understanding of key barriers and facilitators for its implementation in rehabilitation settings.

## 1. Introduction

In the early 1960s, psychologist Carl Rogers used the term patient-centered in psychotherapy to develop the theoretical model called client-centered therapy. This approach aims to understand the patient’s perspective on their problem, through empathy [1]. 

Since then, several authors have considered the importance of integrating psychological issues into this view and developing models aimed at person-centered care (PCC). 

Recently, this view on care has been nominated by health organizations as the model of care to follow. The Institute of Medicine [2] defines it as, ‘providing care that respects and responds to patients’ individual preferences, needs, and values, and ensures that patient’s values guide all clinical decisions’, and suggests inserting PCC as one of the attributes of quality in health.

The World Health Organization [3] states that the care concept should be organized, ‘around people’s health needs and expectations rather than diseases’, and recommends its implementation in all care contexts in both developed and developing countries.

Several authors argue that PCC requires more than individualized care or tailored treatment [4,5]. The PCC framework, developed by McCormack and McCance [6], comprises four constructs: prerequisites focus on the attributes of the healthcare professionals; the care environment focuses on the context in which care is delivered; person-centered processes focus on providing care through a range of activities; and outcomes, the results of effective person-centered care. In addition, this framework has, at its core, the establishment of the therapeutic relationship nurtured in values of mutual respect, understanding, and a person’s right to self-determination.

In the context of rehabilitation, it is essential to have integrated care directed to person-specific needs, meaning that the interventions and approach selected for each person depend on their individual goals and preferences [7].

The essence of PCC focuses on viewing and treating people as self-determining agents, responsible for making their own decisions and playing active roles in their own care [8]. 

PCC can be delivered effectively in patients with long-term conditions, who will greatly benefit from this approach [9].

The development of integrated and PCC systems has the potential to generate significant benefits for people’s health, including improvements in access to care, clinical and health outcomes, health knowledge, and self-care, patient satisfaction, staff satisfaction, the efficiency of services, and a reduction in general costs [3].

Several countries have initiated PCC implementation processes adapted to their health system. However, few have succeeded [10,11]. 

The translation of PCC into practice remains notoriously difficult, and centralization in the person needs to be considered in a broader context. Changing a culture is challenging and laborious. Although there is an increasing emphasis on rehabilitation settings for PCC and the existence of effective interventions, the resistance of professionals and institutions to address this challenge remains [7,12,13].

Carvajal et al. [14] argue that PCC implementation depends on several factors that can act as barriers or facilitators. It is important to understand health professionals’ perceptions to improve the quality of care, as they play an integral role in providing PCC. A better understanding of the factors that impact PCC implementation could improve its delivery. This research explores the rehabilitation workforce’s perspectives on the barriers and facilitators to implementing PCC.

## 2. Methods

The research used a qualitative descriptive design, which is reported following the consolidated criteria for reporting a qualitative research (COREQ) checklist [15].

### 2.1. Participants

The study population consists of the rehabilitation workforce, including physiotherapists, occupational therapists, speech and language therapists, physical medicine and rehabilitation doctors, and rehabilitation nurses. Participants were recruited from two rehabilitation centers from a private institution in the region of Lisbon and Tagus Valley. Recruitment took place in October 2020. A non-probabilistic convenience sample was used, where all eligible healthcare professionals were invited to participate in the study through their institutional e-mail addresses. Of the 14 eligible healthcare professionals, one declined to participate in the study. 

Before the data collection, the researchers discussed and defined saturation as no new barriers or facilitators identified in more than three interviews [16]. The findings of each new interview were compared with those that have already emerged. The first nine interviews were sufficient to identify the themes across the data. The final interviews allowed the researchers to fully understand the barriers and facilitators despite not generating new data. 

### 2.2. Ethics and Procedures

Before conducting this study, a research protocol was approved by the Board of Directors and the Institutional Ethical Review Committee. Prior to the interview, the first author provided a participant information sheet and a verbal explanation of the study. Then, written informed consent to audio-record the interview, anonymously report, and publish the research data was obtained from all participants. Interviews were stored on a password-protected computer, which housed all data. After the verbatim transcription, all the recorded data was destroyed. 

### 2.3. Data Collection

The first author collected data through individual, face-to-face interviews in either a seminar room or a private room at the inpatient unit. No one else was present during the interviews besides the participants and the researcher. To ensure discussion remained pertinent to the aims of the study, an interview guide was developed, comprising a set of open-ended questions focused on the two constructs of barriers and facilitators to PCC. This guide was developed based on the literature review, with input from experts on qualitative research methods, and pilot tested among colleagues.

Examples of questions used in the guide are: Are there any factors limiting you from contributing to PCC? Tell me about a particular example of a barrier or facilitator to PCC? What do you think would assist or facilitate PCC in rehabilitation settings?

On average, each interview lasted approximately 40 min and was audiotaped, transcribed verbatim into textual data by the researchers, anonymized, and later analyzed.

### 2.4. Analysis

Transcribed interviews were imported and coded in QDA Miner Lite database and analyzed using content analysis as described by Braun, Clarke, Hayfield, and Terry [17]. This method focuses on analyzing data to identify recurring common themes, ideas, and patterns of meaning through pre-analysis, encoding, categorization, and interpretation of the data. Two researchers performed this process independently. Codes were assigned to the meaning units using the participants’ own words. Themes and subthemes were developed inductively from the initial codes, reflecting the differences and similarities in the participants’ responses. Afterward, two other researchers reviewed the participant quotes and matched each quote to one of the identified themes. Finally, the whole research team reviewed the final findings.

### 2.5. Trustworthiness

The study’s trustworthiness was confirmed through credibility, transferability, dependability, and confirmability, as Nowell, Norris, White, and Moules [18] described. To enhance credibility, researchers discussed every step made over the analysis process until achieving consensus. Regarding transferability, the researchers provided descriptions with appropriate quotations so that those who sought to transfer the findings to their site could judge transferability. To achieve dependability, researchers detailed every step of the decision-making process so that others could follow the research. Finally, to verify confirmability, external observers searched for inconsistencies by comparing the similarity of their perceptions with those from the researchers. 

## 3. Results

Twelve healthcare professionals participated in the study (Table 1). The participants included rehabilitation nurses (*n* = 5), physiotherapists (*n* = 4), occupational therapists (*n* = 1), speech and language therapists (*n* = 1), and a physical medicine and rehabilitation doctor (*n* = 1). 

Data analysis revealed several barriers to and facilitators of patient-centered care, which we have grouped into subthemes (Table 2). Each subtheme is detailed below.

### 3.1. Barriers to Patient-Centered Care

#### 3.1.1. Unsupportive Organization and Leadership

Several participants described the lack of organizational and leadership values that should serve as role models and forerunners for consistently working in a PCC pathway.

‘*The organizations must align their mission, values, and leadership with patient-centered goals. If staff doesn’t feel that the organization’s targets person-centered care values, they will not carry out patient-centered care!*’ (P1)

#### 3.1.2. Staff Constraints

Participants described that sometimes staff leave was not backfilled, and they could not provide PCC for that reason. They frequently spoke about institutions being below their full-time-equivalent target due to recruitment restraints. This was consistently described as a limitation to PCC implementation.

‘*There are several constraints to PCC. The lack of staff is a major limitation. It is an issue that all healthcare organizations have to deal with. We have staff members absent due to health and personal issues. All institutions struggle to maintain their staff levels.*’ (P4)

#### 3.1.3. Heavy Workload

Participants consistently reported a heavy workload as a barrier toward PCC. This barrier is connected to the subtheme staff constraints. Although participants described good staff–patient ratios, the institution assigned an increasing number of patients to each healthcare professional due to staff leave, and recruitment restraints.

‘*Sometimes the workload is too heavy, and therefore we cannot perform proper rehabilitation care. Is not PCC, is mechanic care aimed for a physical problem.*’ (P8)

#### 3.1.4. Resistance to Change

Participants reported unsupportive staff attitudes as a barrier to PCC, whereby, consciously or unconsciously, they demonstrate a lack of engagement with quality improvement and little motivation for change.

‘*In some staff, there’s an attitude problem. If the organization wants to introduce changes, the staff should consider them and decide what’s best for patients. Maybe it will make our lives easier. Is so there is no need for show resistance to change.*’ (P1)

Several participants reported working according to traditional care pathways, with restrictions to doing things differently to the usual care they administer. This type of care restricts the freedom to engage in PCC.

‘*In rehabilitation for long we provided care based on the biomedical paradigm, It is difficult to change for PCC. You need to change your mentality. People struggle. They resist changing their care.*’ (P11)

#### 3.1.5. Patient’s Clinical Characteristics

The participants performed rehabilitation care for patients with several health problems. Patients possessed varying levels of awareness regarding their condition. Due to this lack of knowledge, they may set goals that might be unrealistic for themselves.

‘*If the patient cannot decide for himself if he has no insight for his illness, it is challenging to carry out patient-centered care.*’ (P5)

### 3.2. Facilitators to Patient-Centered Care

#### 3.2.1. Leadership

For several participants, the leaders served as role models, and the leadership style can instigate organizational change, which could be an enabler for a PCC pathway.

‘*A strong charismatic leader is essential for the development of PCC. You need a strong engagement to get staff on board, and only a strong leader can start this change.*’ (P6)

#### 3.2.2. Staff Satisfaction

Staff satisfaction was frequently reported as a facilitator of PCC. By being satisfied with their work conditions, the rehabilitation workforce will dedicate themselves to pursuing better care and health outcomes for patients.

‘*When everybody is satisfied with their working conditions, it is easier for workers to dedicate themselves to better care. To change care in a healthcare unit, you need to have a team with high levels of satisfaction.*’ (P5)

#### 3.2.3. Positive Physical Environment

Working in a pleasant and relaxing environment that provides comfort and privacy to patients and staff was considered a facilitator of PCC.

‘*If you achieve to create a caring, inclusive and safe environment that inspires rehabilitation professionals to express their vision, creativity and achieve their goals, you will have a team that will pursue PCC. … The right physical environment is also a must-have. For me to provide PCC, I need a private space where I can focus on providing physical comfort as well as emotional well-being.*’ (P2)

#### 3.2.4. Training and Education 

Frequently reported by participants, professional training and education are essential for a successful PCC implementation. For participants, ongoing relevant staff training is vital to reduce healthcare professionals’ knowledge gaps, update their skills, and keep them interested and motivated.

‘*Some of us need training for keeping up with the new methods of care. Our jobs require a commitment to lifelong learning. Training is vital for us to keep our knowledge and skills up-to-date.*’ (P12)

#### 3.2.5. Shared Decision-Making

Participants perceived that shared decision-making facilitates PCC and enables patients to engage with them in the process of care. By sharing the decision-making, the rehabilitation workforce will help patients feel more engaged in their own care and ultimately increase satisfaction with that care.

‘*The patient is the one that ultimately must make the decision. It is their life, and we need to be realistic, help them choose their goals, and support them. The fundamental for the existence of patient-centered care is the patient’s autonomy to decide what’s best.*’ (P9)

## 4. Discussion

The interviews with the rehabilitation workforce identified several PCC barriers and facilitators. In keeping with previous studies, we identified that unsupportive organization and leadership can restrict PCC and undermine the professionals facilitating it. Organizations must support the concept and enable it [19,20]. For PCC to become truly embedded in health organizations more broadly and comprehensively, it must depend on organizational values and culture and not on the behavior of individuals. If an organization fosters a culture of patient-centered care and has leaders who can transmit these values, this will facilitate the real effectiveness of PCC [6,21]. Whether these organizational values and culture actually transferred into facilitating PCC is unknown [13].

In comparison with the results of other research that identified PCC barriers, staff constraints and a heavy workload were also identified [20,22].

To truly enable PCC, the concept must be supported by health care organizations through the development of a conducive environment. The lack of guidelines and role models to champion PCC, staff constraints, heavy workload, lack of teamwork, and the high nurse–patient ratio, have been associated with poor nurse retention rates, low patient satisfaction and outcomes [21,22]. These organizational barriers can impact the effectiveness of PCC [14,21,22].

The barrier, resistance to change, emerged from the analysis related to traditional practices and staff attitudes. The rehabilitation workforce that follows traditional care pathways, practices their care based on the biomedical paradigm. With the restriction to a patients’ freedom to be involved in shared decision-making, it is challenging to obtain significant changes. A flexible practice of care is described by some researchers as a prerequisite for PCC [6,20]. Previous researchers also identified resistance to change as a barrier to PCC. They reported findings of paternalistic practices, professionals prioritizing objectives and ‘medicalized’ interventions, and lack of motivation [13,21].

Importantly, this study identified a new barrier to PCC. Participants report that the patient’s clinical characteristics influence the development of PCC. Despite not being identified in previous studies reporting PCC barriers and facilitators, several authors have highlighted the need to investigate these factors in different settings. Although we did not find any study that corroborates this evidence, considering that a key element of PCC is establishing a therapeutic relationship, it is expected that the characteristics of both parties in relation to each other, act as possible barriers or facilitators.

Regarding the PCC facilitators, in addition to leadership, which has already been a focus throughout this discussion, staff satisfaction, a positive physical environment, training and education, and shared decision-making, were also facilitators reported by participants. 

Several factors related to the care environment have been linked to a positive or negative effect on the promotion of PCC [6,13,21]. For example, in the research of Lloyd et al. [20] and Morera-Balaguer et al. [23], the environment constraints were described by participants as a barrier to PCC. They also reported a lack of spaces that provide privacy to establish a relationship of trust and confidence, which is central to PCC. 

Several researchers have found evidence that there is a link between staff satisfaction and PCC [20,24]. When comparing the barriers and facilitators identified in this study, we found that many of the barriers were directly linked to decreased staff satisfaction, so it seems logical that participants have identified this facilitator.

Healthcare is typified by change. These changes can increase stress, decrease job satisfaction, and create resistance to change [25]. A rehabilitation workforce with higher levels of resilience and satisfaction is more receptive to committing to organizational change, and given that PCC is a radical change from the biomedical paradigm [24,25], this is an important factor to be considered

The ultimate goal of PCC is to help patients play an active role in the decisions that concern their health [6,23]. Shared decision-making enables patients to engage in the PCC process. Participants frequently reported this factor because they felt it was the first step towards PCC. This facilitator is a key to establishing a therapeutic relationship and stimulates the patient to play an active role in their treatment [26].

Training and education are essential prerequisites for professional competence and, therefore, educational programs that allow the development of nursing skills to provide holistic care were identified as being a relevant facilitator for PCC [14,27,28].

The objective of this research was to explore the rehabilitation workforce perspectives on the barriers and facilitators to implementing PCC. PCC is sensitive to the specific care context, but this research identified barriers and facilitators that were also important factors impacting its implementation in other settings. Given this finding, this research supports the idea that interventions outlined to target these factors, may assist healthcare providers in achieving PCC in different settings.

There are few studies concerning the barriers and facilitators to PCC in rehabilitation settings. Therefore, we recommend developing more empirical studies regarding this research area.

### Limitations

As identified in previous studies reliant on data collected from interviews, there is a possibility that actual reports were different from what participants disclosed. This could be due to biases that influence the information reported by participants, such as lack of confidence in ensuring anonymity or protection of identity and professional values or beliefs. However, considering that data were identified from several participants, we believe it is unlikely that this occurred. Another limitation is its transferability, as convenience sampling from one institution represents greater operational ease but limits the ability to make general statements about the study results. Finally, the sample size was small (*n* = 12); however, it was enough for no new themes to emerge.

PCC has the potential to transform rehabilitation workforce clinical practice, but there must be several factors in place. Further research is needed to better understand the relationships and impact of these factors on PCC.

## 5. Conclusions

PCC practice can produce significant benefits for people’s health. However, its application remains extremely difficult. Therefore, it is essential to understand the rehabilitation workforce perceptions, as these workers play an integral role in providing PCC. This study serves as a guide to facilitate PCC, as it allows for an understanding of the key barriers to and facilitators of its implementation in rehabilitation settings. Participants reported different barriers, such as staff constraints, heavy workload, staff experience, resistance to change, an unsupportive organization and leadership, and patients’ clinical characteristics. As facilitators, they described training and education, leadership, staff satisfaction, a positive physical environment, and shared decision-making.

Different barriers and facilitators were identified and showed that not only staff behavior and their characteristics influence its implementation. In other research, several additional factors were identified as influencing PCC. Unique to this study, a patient’s knowledge of their own clinical characteristics was identified as a barrier to PCC.

## Figures and Tables

**Table 1:** Characteristics of the participants.

Characteristics		Frequency	Percentage
Sex	Woman	8	66.7
	Man	4	33.3
Age	30–40	6	50
	40–50	4	33.3
	50–60	2	16.7
Profession	Rehabilitation nurses	5	41.8
	Physiotherapists	4	33.3
	Occupational therapists	1	8.3
	Speech and language therapists	1	8.3
	Physical medicine and rehabilitation doctors	1	8.3

**Table 2:** Barriers and facilitators to patient-centered care.

Themes	Subthemes	Participants (*N* = 12)
Barriers to Patient-centered Care	Unsupportive organization and leadership	11
	Staff constraints	10
	Heavy workload	10
	Resistance to change	9
	Patient’s clinical characteristics	7
Facilitators to Patient-centered Care	Leadership	10
	Staff satisfaction	10
	Positive physical environment	9
	Training and education	8
	Shared decision-making	6

## Data Availability

The data presented in this study are available on request from the first author.

## References

[1] The Health Foundation (2016). Person-Centred Care Made Simple.

[2] Institute of Medicine (2001). Crossing the Quality Chasm: A New Health System for the 21st Century.

[3] World Health Organization (2016). Framework on Integrated, People-Centred Health Services.

[4] El-Alti L., Sandman L., Munthe C. (2019). Person Centered Care and Personalized Medicine: Irreconcilable Opposites or Potential Companions?. Health Care Anal..

[5] Santana M.J., Manalili K., Jolley R.J., Zelinsky S., Quan H., Lu M. (2018). How to practice person-centred care: A conceptual framework. Health Expect..

[6] McCormack B., McCance T. (2017). Person-Centred Practice in Nursing and Health Care: Theory and Practice.

[7] World Health Organization (2021). Rehabilitation. https://www.who.int/news-room/fact-sheets/detail/rehabilitation.

[8] Davidson L., Tondora J., Miller R., O’Connell M.J., Corrigan P.W. (2015). Person-centered care. Person-Centered Care for Mental Illness: The Evolution of Adherence and Self-Determination.

[9] Eaton S. (2016). Delivering person-centred care in long-term conditions. Future Hosp. J..

[10] Rahman R., Matthews E.B., Ahmad A., Rizvi S.M., Salama U., Samad L., Khan M. (2019). Perceptions of patient-centred care among providers and patients in the orthopaedic department of a tertiary care hospital in Karachi, Pakistan. J. Eval. Clin. Pract..

[11] Sharp S., McAllister M., Broadbent M. (2016). The vital blend of clinical competence and compassion: How patients experience person-centred care. Contemp. Nurse.

[12] Fernandes J., Fernandes S., Almeida A., Vareta D., Miller C. (2021). Older Adults’ Perceived Barriers to Participation in a Falls Prevention Strategy. J. Pers. Med..

[13] Moore L., Britten N., Lydahl D., Naldemirci Ö., Elam M., Wolf A. (2017). Barriers and facilitators to the implementation of person-centred care in different healthcare contexts. Scand. J. Caring Sci..

[14] Carvajal A., Haraldsdottir E., Kroll T., McCormack B., Errasti-Ibarrondo B., Larkin P. (2019). Barriers and facilitators perceived by registered nurses to providing person-centred care at the end of life. A scoping review. Int. Pract. Dev. J..

[15] Tong A., Sainsbury P., Craig J. (2007). Consolidated criteria for reporting qualitative research (COREQ): A 32-item checklist for interviews and focus groups. Int. J. Qual. Health Care.

[16] Hennink M., Kaiser B.N. (2022). Sample sizes for saturation in qualitative research: A systematic review of empirical tests. Soc. Sci. Med..

[17] Braun V., Clarke V., Hayfield N., Terry G., Liamputtong P. (2019). Thematic analysis. Handbook of Research Methods in Health Social Sciences.

[18] Nowell L.S., Norris J.M., White D.E., Moules N.J. (2017). Thematic Analysis: Striving to Meet the Trustworthiness Criteria. Int. J. Qual. Methods.

[19] Deek H., Hamilton S., Brown N., Inglis S.C., Digiacomo M., Newton P.J., Noureddine S., MacDonald P.S., Davidson P.M., FAMILY Project Investigators (2016). Family-centred approaches to healthcare interventions in chronic diseases in adults: A quantitative systematic review. J. Adv. Nurs..

[20] Lloyd B., Elkins M., Innes L. (2018). Barriers and enablers of patient and family centred care in an Australian acute care hospital: Perspectives of health managers. Patient Exp. J..

[21] Kiwanuka F., Shayan S., Tolulope A. (2019). Barriers to patient and family-centred care in adult intensive care units: A systematic review. Nurs. Open.

[22] Esmaeili M., Cheraghi M., Salsali M. (2014). Barriers to patient-centered care: A thematic analysis study. Int. J. Nurs. Knowl..

[23] Morera-Balaguer J., Botella-Rico J., Catalán-Matamoros D., Martínez-Segura O., Leal-Clavel M., Rodríguez-Nogueira Ó. (2019). Patients’ experience regarding therapeutic person-centered relationships in physiotherapy services: A qualitative study. Physiother. Theory Pract..

[24] Huang C.Y., Weng R.H., Wu T.C., Hsu C.T., Hung C.H., Tsai Y.C. (2020). The impact of person-centred care on job productivity, job satisfaction and organisational commitment among employees in long-term care facilities. J. Clin. Nurs..

[25] Brown R., Wey H., Foland K. (2018). The Relationship Among Change Fatigue, Resilience, and Job Satisfaction of Hospital Staff Nurses. J. Nurs. Scholarsh..

[26] Brooks L., Manias E., Nicholson P. (2017). Barriers, enablers and challenges to initiating end-of-life care in an Australian intensive care unit context. Aust. Crit. Care.

[27] Dong F., Zheng R., Chen X., Wang Y., Zhou H., Sun R. (2016). Caring for dying cancer patients in the Chinese cultural context: A qualitative study from the perspectives of physicians and nurses. Eur. J. Oncol. Nurs..

[28] Ranse K., Yates P., Coyer F. (2016). End-of-life care practices of critical care nurses: A national cross-sectional survey. Aust. Crit. Care.

